# Role and Regulation of the Flp/Tad Pilus in the Virulence of *Pectobacterium atrosepticum* SCRI1043 and *Pectobacterium wasabiae* SCC3193

**DOI:** 10.1371/journal.pone.0073718

**Published:** 2013-09-09

**Authors:** Johanna Nykyri, Laura Mattinen, Outi Niemi, Satish Adhikari, Viia Kõiv, Panu Somervuo, Xin Fang, Petri Auvinen, Andres Mäe, E. Tapio Palva, Minna Pirhonen

**Affiliations:** 1 Department of Agricultural Sciences, University of Helsinki, Helsinki, Finland; 2 Department of Biosciences, University of Helsinki, Helsinki, Finland; 3 Institute of Molecular and Cell Biology, University of Tartu, Tartu, Estonia; 4 Institute of Biotechnology, University of Helsinki, Helsinki, Finland; University of Wisconsin-Milwaukee, United States of America

## Abstract

In this study, we characterized a putative Flp/Tad pilus-encoding gene cluster, and we examined its regulation at the transcriptional level and its role in the virulence of potato pathogenic enterobacteria of the genus *Pectobacterium*. The Flp/Tad pilus-encoding gene clusters in *Pectobacterium atrosepticum*, *Pectobacterium wasabiae* and *Pectobacterium aroidearum* were compared to previously characterized *flp*/*tad* gene clusters, including that of the well-studied Flp/Tad pilus model organism *Aggregatibacter actinomycetemcomitans*, in which this pilus is a major virulence determinant. Comparative analyses revealed substantial protein sequence similarity and open reading frame synteny between the previously characterized *flp*/*tad* gene clusters and the cluster in *Pectobacterium*, suggesting that the predicted *flp*/*tad* gene cluster in *Pectobacterium* encodes a Flp/Tad pilus-like structure. We detected genes for a novel two-component system adjacent to the *flp*/*tad* gene cluster in *Pectobacterium*, and mutant analysis demonstrated that this system has a positive effect on the transcription of selected Flp/Tad pilus biogenesis genes, suggesting that this response regulator regulate the *flp*/*tad* gene cluster. Mutagenesis of either the predicted regulator gene or selected Flp/Tad pilus biogenesis genes had a significant impact on the maceration ability of the bacterial strains in potato tubers, indicating that the Flp/Tad pilus-encoding gene cluster represents a novel virulence determinant in *Pectobacterium*. Soft-rot enterobacteria in the genera *Pectobacterium* and *Dickeya* are of great agricultural importance, and an investigation of the virulence of these pathogens could facilitate improvements in agricultural practices, thus benefiting farmers, the potato industry and consumers.

## Introduction

Soft-rot enterobacteria of the genera *Pectobacterium* and *Dickeya* are devastating phytopathogens that exert significant negative impacts on agricultural production, causing losses in fields and in storage. Many taxonomic groups of soft-rot enterobacteria have been isolated from potato (*Solanum tuberosum* L.), which represents one of the most economically important hosts of these pathogens. These bacteria include *Pectobacterium atrosepticum*, *Pectobacterium carotovorum* subsp. *brasiliensis*, a clade of *Pectobacterium wasabiae* and a clade of *Dickeya* that is now tentatively known as *Dickeya solani* [1], [2], [3], [4], [5], [6]. In addition to their agricultural importance, the investigation of plant pathogenic enterobacteria could also benefit studies of animal pathogens due their kinship with well-known animal pathogens in the Enterobacteriaceae family such as *E. coli*, *Salmonella* and *Yersinia*. This study could provide information that is relevant to other bacterial groups through the investigation of conserved virulence-related systems present in a variety of pathogens.

Soft-rot enterobacteria are necrotrophs, and their pathogenesis relies on suitable environmental conditions that support the multiplication of these opportunistic pathogens and their prolific production of plant cell wall-degrading enzymes (PCWDEs), which cause the typical symptoms of soft rot [3]. In addition to PCWDEs, soft-rot enterobacteria utilize several other factors to colonize plant tissue and enhance disease progression. Such factors include the extracellular Nip (necrosis inducing protein) and Svx (a protein similar to an avirulence protein in *Xanthomonas*) proteins in *Pectobacterium*, intracellular effectors secreted into the host cell via the type III secretion system (T3SS) that have been characterized in both *Pectobacterium* and *Dickeya* and type IV and VI secretion systems (T4SS, T6SS) in *Pectobacterium* [5], [6]. However, T3SS is not present in all *Pectobacterium* and *Dickeya* strains [5], [6]. In addition, motility and surface structures such as lipopolysaccharide (LPS) are important virulence determinants that facilitate the persistence of the bacteria and colonization of the host plant [7], [5]. In *Dickeya*, siderophores, which function in iron acquisition from the surroundings play a major role in virulence; however, their role has not yet been described in the *Pectobacterium* genus [5]. In *Pectobacterium*, the production of PCWDEs and other virulence determinants, such as T3SS and T6SS, is regulated by a complex network in which several virulence traits are controlled by the same regulatory systems [5], [8], [9].

We previously showed that *Pectobacterium* may have a novel uncharacterized putative virulence determinant, the Flp/Tad pilus, which is encoded by the *flp*-*tad*-*rcp* (fimbrial low-molecular-weight protein/tight adherence protein/rough colony protein) gene cluster (also referred to as the *flp*/*tad* gene cluster). This gene cluster was expressed parallel to known virulence determinants such as PCWDEs and T6SS in response to potato tuber extract in *P. atrosepticum* SCRI1043 [10]. The Flp/Tad pilus has been categorized as a type IVb pili, and the encoding cluster is present in a wide variety of bacterial species and is considered a target of horizontal gene transfer [11], [12]. To our knowledge, no type IV pili are related to virulence in soft-rot enterobacteria. The Flp/Tad pilus is composed of Flp/Fap pilin component proteins (fimbrial low-molecular-weight protein/fibril-associated protein), and the pilus often exhibits polar localization on the surface of bacteria. At a minimum, the *flp*/*tad* gene cluster encodes the Flp/Tad pili and the proteins necessary for the biogenesis of these pili [13], [14], [15], [16], [17]. The Flp/Tad pilus was first characterized in *Aggregatibacter actinomycetemcomitans*, the causative agent of localized aggressive periodontitis, in which the pilus is essential for colony morphology and biofilm formation and functions as an important virulence factor (reviewed most recently by Tomich et al. [11]). Since then, the Flp/Tad pilus has been characterized in several other animal pathogenic bacteria of the genera *Haemophilus*, *Pasteurella*, *Pseudomonas* and *Yersinia* and in one environmental bacterium of the genus *Caulobacter* and has been shown to be necessary for biofilm formation and/or virulence [11]. The Flp/Tad pilus is also an important host colonization factor in the gut bacterium *Bifidobacterium breve* [16]. The Flp/Tad pilus locus was recently shown to encode a novel virulence determinant in a phytopathogen (*Ralstonia solanacearum*), indicating for the first time an important role for the pilus in other plant pathogenic bacteria as well [17].

In this study, we further characterized *flp*/*tad* genes encoding the predicted Flp/Tad pilus in soft-rot enterobacteria and examined their role and regulation in virulence. First, we performed a comparative genomics analysis of the *flp*/*tad* gene cluster and identified a conserved cluster among soft-rot enterobacteria similar to that in other bacterial species. We determined that the genes in the *flp*/*tad* gene cluster may be regulated by a novel two-component system (TCS) in soft-rot enterobacteria. Furthermore, we were able to demonstrate that mutagenesis of either selected *flp*/*tad* genes or the novel response regulator of the TCS delayed tissue maceration in potato tubers compared with the wild-type strain. The novel response regulator identified in this work may be an independent part of the regulatory web of virulence in soft-rot enterobacteria and mainly regulates the *flp*/*tad* gene cluster in response to environmental cues similar to those used by other virulence determinants. This study provides novel information regarding virulence determinants in soft-rot enterobacteria, providing a foundation for applied studies aimed at improving plant health, an economically important aspect of agricultural production and industry.

## Materials and Methods

### Bacterial Strains and Standard Culture Conditions

In this study, *Pectobacterium atrosepticum* SCRI1043 [18], *Pectobacterium wasabiae* SCC3193 [19] and their derivatives (Table S1) were utilized as bacterial models, and potato cv. Van Gogh (H&H Tuominen, Finland) was used as a plant model. *Escherichia coli* DH5α was utilized for molecular cloning. *Pectobacterium* strains were grown under standard conditions in Luria broth (L3522, Sigma-Aldrich) for 1 d at 28°C, and *E. coli* was grown in Luria broth for 1 d at 37°C.

### Bioinformatic Tools for Comparative Genomics

To identify the *flp*/*tad* gene cluster and compare the presence and organization of the gene cluster in different bacterial genomes, protein sequences were retrieved from the NCBI database and utilized for comparison via blastp [20], [21]. The nucleotide sequences of the *flp*/*tad* gene clusters in *Pectobacterium* and *Dickeya* were also compared by utilizing blastn to search against the nucleotide collection (nr/nt) and whole genome shotgun contig databases in GenBank of NCBI (http://blast.ncbi.nlm.nih.gov/Blast.cgi). To characterize the missing open reading frame (ORF) of the Flp/Fap pilin component in *P. atrosepticum* SCRI1043, selected genomic sequence of the *flp*/*tad* gene cluster of *P. atrosepticum* SCRI1043 was analyzed using the ORF finder of NCBI (http://www.ncbi.nlm.nih.gov/gorf/gorf.html), and the predicted ORF was confirmed by comparison with close relatives in the genus *Pectobacterium* by sequence alignment utilizing blastn [20], [21] and Clustal Omega sequence alignment programs. Clustal Omega is available at http://www.ebi.ac.uk/Tools/msa/clustalo/.

### Mutagenesis and *in trans* Complementation

The λRED recombinase system [22] was utilized for mutagenesis. For homologous recombination, an antibiotic cassette was amplified from a template plasmid (pKD3) using specific primers with sequence similarity to the template (P1 or P2 site) and to the target sequence in the bacterial genome (Table S1). The antibiotic cassette was amplified using the proofreading PCR enzyme Phusion (F-530, Thermo Scientific/Finnzymes, Finland), and the product was gel purified. Transformation and homologous recombination by λRED were performed for *Pectobacterium* as previously described [10], [6]. To complement genomic mutants, the target gene or genes were amplified by PCR utilizing gene-specific primers (Table S1) and the proofreading enzyme Phusion (F-530, Thermo Scientific/Finnzymes, Finland) according to the manufacturer’s instructions. The PCR products of ECA0785 (Flp/Tad response regulator) and ECA3435 (VasH, sigma54-dependent transcriptional activator) were gel purified, digested with BamHI-SacI and HindIII-SacI (HF enzymes; NEB), respectively, and ligated (T4 ligase; NEB) into the transcription vector pMW119 (Nippon Gene Co., Japan). The PCR product of W5S_0783 (Flp/Fap pilin component) was also gel purified, digested with HindIII-SacI (FastDigest; Thermo Scientific) and ligated (T4 DNA ligase; Thermo Scientific) into pMW119.

### Gene Expression Studies Utilizing Microarray and qPCR

Microarray sample (n = 3) preparation and the microarray procedure, including statistical analyses, were performed as described in our previous work [10]. For gene expression studies by relative qPCR, bacteria were cultured until late log phase in Luria broth or *hrp*-inducing minimal medium salts supplemented with 0.4% polygalacturonic acid (PGA, P3850; Sigma-Aldrich) or 10% v/v potato tuber extract at 15°C (for microarray validation) or 28°C (for examining *flp*/*tad*-related genes). The growth curves were measured under the same conditions and independently repeated 3 times with 3 replicates in each experiment. Bacterial cells were harvested at late log/early stationary phase, and total RNA was extracted as described earlier [23]. Prior to DNAse treatment (Ambion TURBO DNA-free™ Kit) and cDNA synthesis (Invitrogen VILO), the RNA was purified using the Qiagen RNA cleanup kit. RNA concentration and integrity were analyzed by agarose gel electrophoresis and spectrophotometric measurements. For qPCR (Roche LightCycler® 480 Real-Time PCR System), 3 technical replicates were performed for each sample, and each reaction (LightCycler® 480 SYBR Green I Master) contained 100 ng cDNA. The results were normalized from Cp values by utilizing a previously described reference gene *proC* [24], [10] and 2^−ΔΔCT^-method [25]. Primers for genes tested by qPCR can be found in Table S1. Statistical analyses were performed utilizing Student’s t-test function in Excel (TTEST, Microsoft Office) as a pairwise comparison of selected bacterial strains (n = 3, independent biological replicates per strain).

### Accession Number of the Microarray Experiment

The microarray data discussed in this publication have been deposited in NCBI’s Gene Expression Omnibus [26] and are accessible through GEO Series accession number GSE48471 (http://www.ncbi.nlm.nih.gov/geo/query/acc.cgi?acc=GSE48471).

### Virulence Assays on Potato Tuber Slices

Bacteria were grown overnight and washed once with 10 mM MgSO_4_ buffer, resuspended in the same buffer and adjusted to an OD_600_ of 0.26 (SCRI1043) or 1.6 (SCC3193). Potato tubers (cv. Van Gogh) were washed with tap water, surface sterilized in Na-hypochlorite for 7 min, washed 4 times with sterile deionized water and air-dried. Based on preliminary experiments, the virulence assay settings were optimized separately for both wild-type strains to analyze how mutations affect the virulence of the bacterial strains. The tubers were labeled and stabbed with a pipette tip to create a cavity for bacterial inoculation (50 µl of SCRI1043 and its derivatives, 10 µl of SCC3193 and its derivatives or 10 mM MgSO_4_). The wounds were sealed with white Vaseline (YA, Finland), and the inoculated tubers were wrapped in wet paper tissues and plastic wrap, placed into a plastic seedling box, covered with a lid and sealed with masking tape. The tubers were incubated at room temperature in the shade for 5 days (for SCRI1043 and its derivatives) or 3 days (for SCC3193 and its derivatives). After incubation, the potato tubers were cut in half, and the softened tissue was scraped out and weighed. Statistical analyses were performed for each independent experiment (n = 10–15 tubers per strain) using Student’s t-test (equal variance) function in Excel (TTEST, Microsoft office). Similar results were obtained from a minimum of 3 independent experiments.

### Enzymatic Assays

Assays to detect PCWDE production were conducted according to previous publications [27]. Bacteria were grown overnight on Luria broth plates, and fresh colonies were stabbed into indicator plates to determine cellulase and pectinase secretion. *P. wasabiae* SCC3193 was grown on indicator plates for 1 d at 28°C, and *P. atrosepticum* was incubated on indicator plates for 2 d at 28°C. The diameters of the halos that formed around the bacteria due to substrate utilization were measured. The experiments were repeated 3 times.

### Motility Assays

For motility assays, plates containing *hrp*-inducing minimal medium salts, 0.4% PGA and 0.25% agar were prepared, and colonies from fresh bacterial plates were stabbed into the agar. The plates were incubated overnight at room temperature, after which the dispersion of bacteria was assessed and the plates were photographed. Motility assays were performed a minimum of 3 times.

### Biofilm Formation Assay in Polypropylene Eppendorf Tubes

Biofilm formation was assessed essentially as described by O’Toole and Kolter [28]. Overnight bacterial cultures grown in Luria broth (10 µl) were inoculated into 400 µl Luria broth or M9 minimal salts supplemented with 0.4% glycerol as the sole carbon source in polypropylene tubes (Eppendorf). After 6 and 18 h of incubation at 30°C, 70 µl of 1% (w/v) crystal violet solution was added to each tube and incubated at room temperature for 20 min. After washing the tubes 3 times with distilled water, 600 µl 96% ethanol was added to each tube to extract crystal violet from the cells. Aliquots (100 µl) of crystal violet in ethanol were quantified in a microtiter plate at A_540_ using an ELISA reader (Tecan Sunrise-Basic). Each assay was performed at least 3 times with 3–5 parallel samples for each variant.

## Results

### The Flp/Tad Pilus-encoding Gene Cluster is Commonly Present in *Pectobacterium*

**Figure 1:**
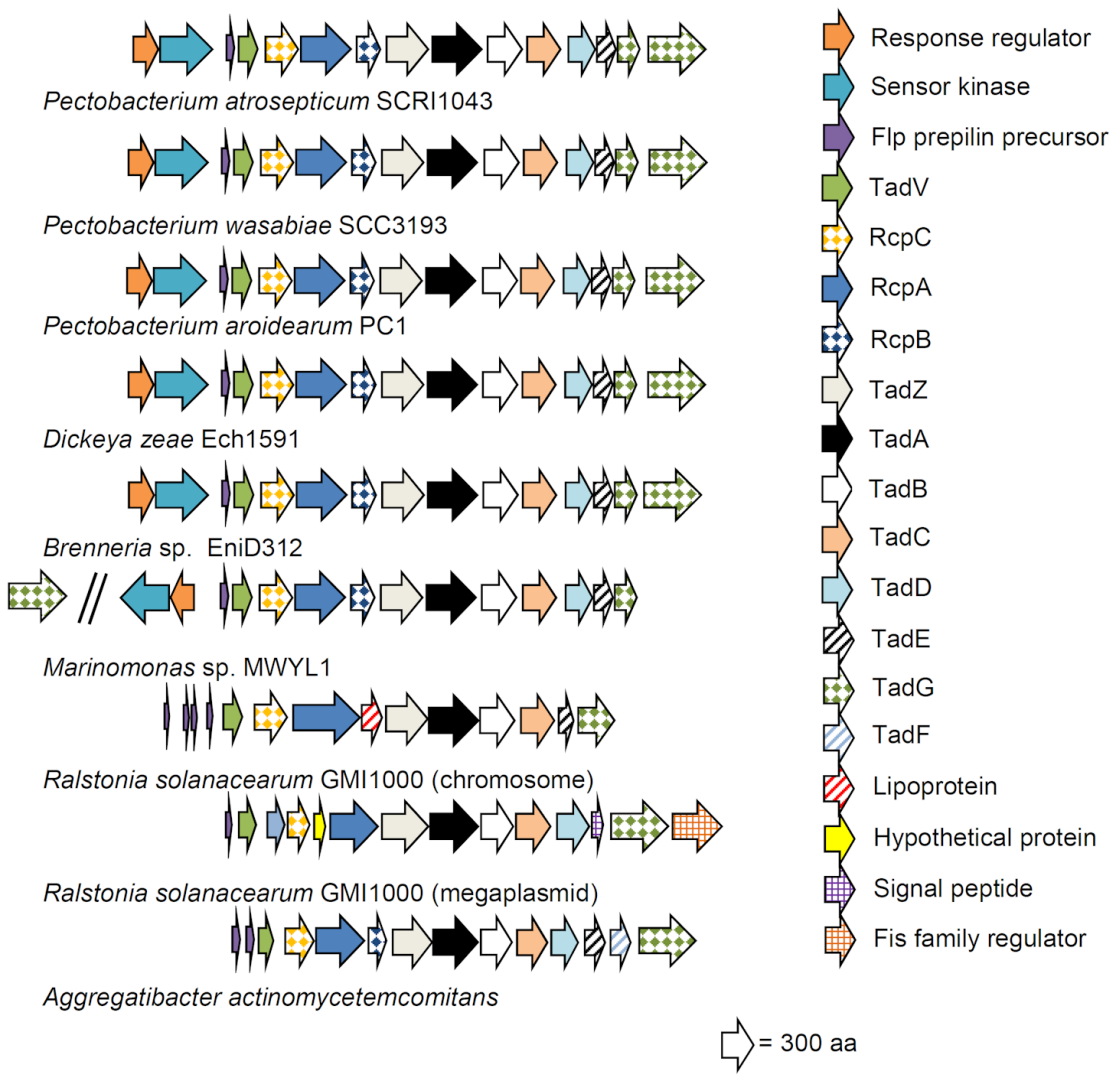
Predicted Flp/Tad pilus-encoding gene cluster in *Pectobacterium*. Comparative genomics analysis revealed that the synteny of the gene clusters encoding the putative novel virulence determinant Flp/Tad pilus in *Pectobacterium* and in one *Dickeya* species is similar to that in the well-studied Flp/Tad model species *Aggregatibacter actinomycetemcomitans*. Flp = fimbrial low-molecular-weight protein. Tad = tight adherence protein. Rcp = rough colony protein. The gene cluster comparison was based on genomic and protein sequence comparisons utilizing blastn and blastp.

Comparative genomics analyses demonstrated that the putative Flp/Tad-like pilus-encoding gene cluster is conserved in all *Pectobacterium* species for which genome sequences are available in GenBank (*P. carotovorum*, *P. atrosepticum*, *P. wasabiae*, *P. aroidearum* and *P. carotovorum* subsp. *brasiliensis*); however, this cluster is only conserved in one *Dickeya* strain. In addition, its structure and/or synteny are highly similar to those of Flp/Tad-like clusters previously characterized in several other species (Figure 1). We also discovered a predicted novel regulator (TCS) adjacent to the *flp*/*tad* gene cluster in soft-rot enterobacteria (Figure 1). In one of our model strains, *P. atrosepticum* SCRI1043, one of the necessary Flp/Tad pilus-encoding genes (Flp/Fap pilin component) is not predicted as an ORF [29]; however, based on our revised ORF analysis, a gene is present (start-stop; 861131–861352) in the same position with high sequence similarity (query coverage 100%, identity 91–94%) to other *Pectobacterium* species for which complete genome information is available (Figure S1).

The *flp*/*tad* gene cluster in soft-rot enterobacteria is highly similar (on the amino acid level) to clusters with the same synteny in *Brenneria* sp. EniD312 of the Enterobacteriaceae (for example the Flp/Fap pilin component has a query coverage of 79% and an identity of 90% compared to SCRI1043) and *Marinomonas* sp. MWYL1 of the Oceanospirillaceae (for example the Flp/Fap pilin component has a query coverage of 75% and an identity of 79% compared to SCRI1043), which are phylogenetically more distant members of the Gammaproteobacteria compared with soft-rot enterobacteria (Figure 1). In addition, the synteny of the well-studied Flp/Tad pilus-encoding cluster in *A*. *actinomycetemcomitans* is similar to that of the cluster in soft-rot enterobacteria; however, the amino acid sequence similarity is low (for example the *Aggregatibacter* Flp/Fap pilin component BAA25886 has a query coverage of 70% and an identity of 42% compared to SCRI1043) compared with that of the soft-rot enterobacteria *Brenneria* and *Marinomonas* (Figure 1). The *flp*/*tad* gene clusters in *R. solanacearum* were characterized previously by Wairuri and colleagues [17]. Interestingly, the organization of the *flp*/*tad* gene clusters in the plant pathogenic bacterium *Ralstonia solanacearum* differ slightly and their sequence similarity is distinct from that of soft-rot enterobacteria (Figure 1). The blastx alignment was not useful (result: no significant similarity was found) for comparing *P. atrosepticum* SCRI1043 (Flp/Fap pilin component start-stop; 861131–861352) and *R. solanacearum* GMI1000 (Flp/Fap pilin component; RSc0659) or *A*. *actinomycetemcomitans* (Flp/Fap pilin component; BAA25886) and *R. solanacearum* GMI1000 (genomic Flp/Fap pilin component; RSc0659).

The marked sequence and/or synteny similarity of the *flp*/*tad* gene cluster in *Pectobacterium* to that of distantly related bacteria and the lack of the *flp*/*tad* gene cluster in some close relatives suggest that the predicted *flp*/*tad* gene cluster in soft-rot enterobacteria indeed encodes the Flp/Tad pilus and that the locus is likely of horizontal origin, benefiting several bacterial species independent of their lifestyle.

### A Novel Two-component System Regulates Flp/Tad Pilus-encoding Genes in *Pectobacterium*

**Figure 2:**
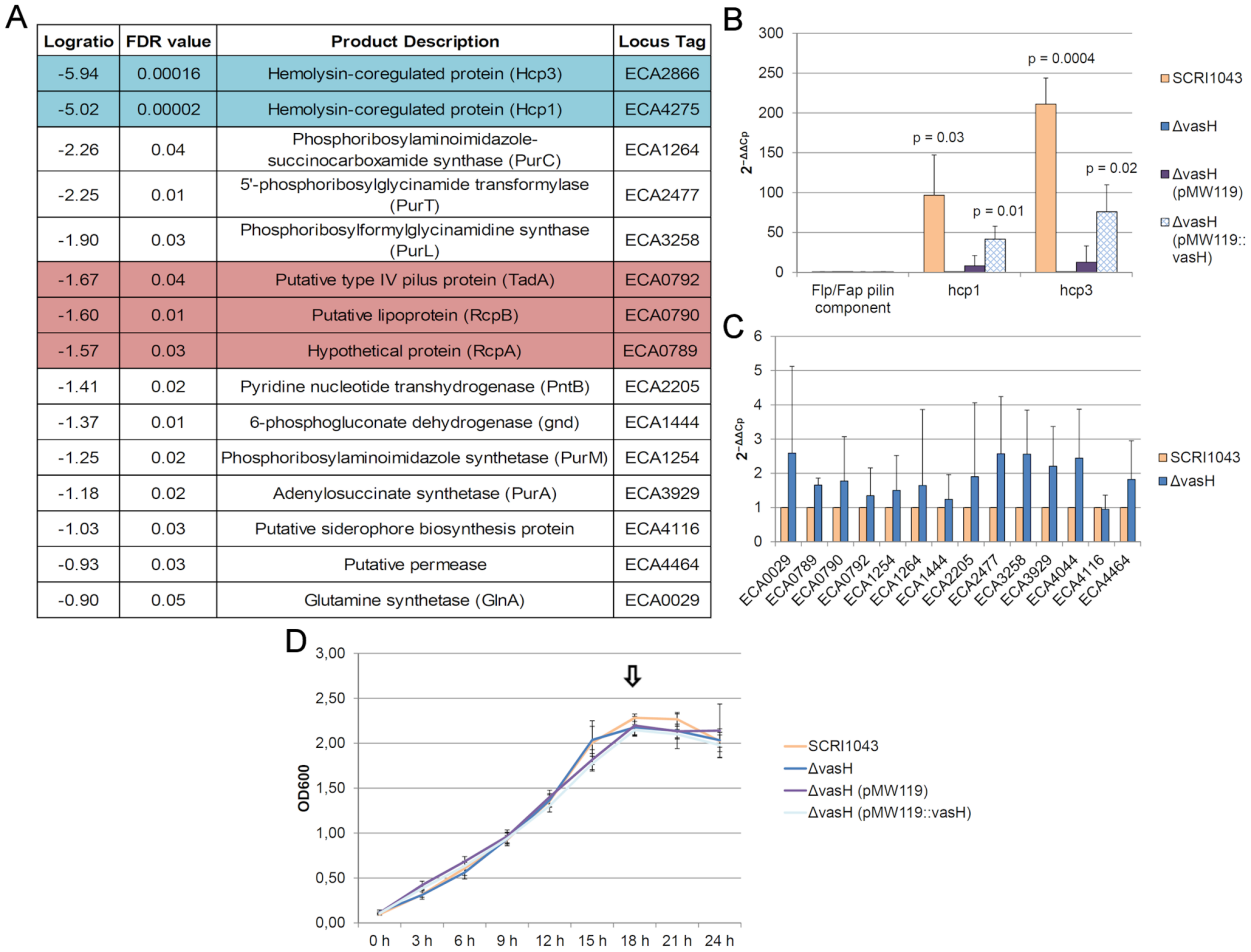
VasH regulates *hcp* genes but not *flp*/*tad* genes in *Pectobacterium atrosepticum*. A) Microarray data showing that 15 genes were downregulated in the Δ*vasH* mutant (FDR<0.05) compared with the wild-type strain *P. atrosepticum* SCRI1043. The two T6SS-related Hcp-encoding genes are marked with blue, and the three Flp/Tad pilus-related genes are marked with red. The microarray results represent the average of three independent experiments. B) Relative qPCR validation of the microarray results and complementation of the mutant *vasH* (ECA3435) *in trans* indicate that VasH is likely to regulate *hcp* genes (p<0.03) but is not likely to regulate the predicted Flp/Fap pilin component-encoding gene (2^−ΔΔCt^: SCRI1043; 0.7, Δ*vasH*; 1, Δ*vasH*(pMW119); 0.5, Δ*vasH* (pMW119::*vasH*); 0.7, p value for all relevant comparisons >0.08) in *P. atrosepticum* SCRI1043 C) or other *flp*/*tad* genes observed in the microarrays (ECA0789, ECA0790 and ECA0792) or any other genes that were downregulated in the microarrays. ECA4044 is a negative control and was not differentially expressed in the microarrays. The qPCR experiments were repeated a minimum of three times, and the graphs show the averages and standard deviations of three independent experiments. D) Growth of *P. atrosepticum* SCRI1043 and Δ*vasH* in *hrp*-inducing minimal medium salts supplemented with 10% v/v potato tuber extract at 15°C. The sampling point for the microarray and qPCR experiments is marked with an arrow. The growth curves show the averages and standard deviations of three replicates in a single experiment, which was repeated a minimum of three times with similar results.

We previously demonstrated that *flp*/*tad* genes were upregulated in the same plant mimicking condition as T6SS-related genes [10]. In this study, we wanted to more closely investigate genes regulated by the T6SS-related sigma54-dependent transcriptional activator (VasH) and examine the cross-regulation of T6SS and the Flp/Tad pilus in *P. atrosepticum* SCRI1043 utilizing microarray technology. VasH is a regulator of the *hcp* and *vgrG* genes, which are related to T6SS [30], [31]. However, of the statistically significant (FDR<0.05) differentially expressed genes in the microarrays (including three *flp*/*tad* genes) (Figure 2A, Figure 2D), only T6SS-related *hcp* genes were complemented *in trans* in the VasH mutant as assessed by the more sensitive relative qPCR method, as shown in independent experiments (Figure 2B, Figure 2C, Figure 2D). Further investigations are necessary to reveal the regulation of the *flp*/*tad* gene cluster in *Pectobacterium*.

Subsequently, we examined the role of the TCS that we discovered (Figure 1) adjacent to the predicted Flp/Tad pilus-encoding gene cluster (in *P. atrosepticum* SCRI1043, ECA0785-ECA0786; in *P. wasabiae* SCC3193, W5S_0781-W5S_0782) in the transcription of *flp*/*tad* genes. The predicted proteins of these gene pairs have typical features of TCS, such as an OmpR (COG0745) domain, a REC domain of response regulators (in ECA0785 and W5S_0781), an ATPase domain, a BaeS kinase (COG0642) domain and histidine kinase domains of a histidine kinase sensor (in ECA0786 and W5S_0782).

**Figure 3:**
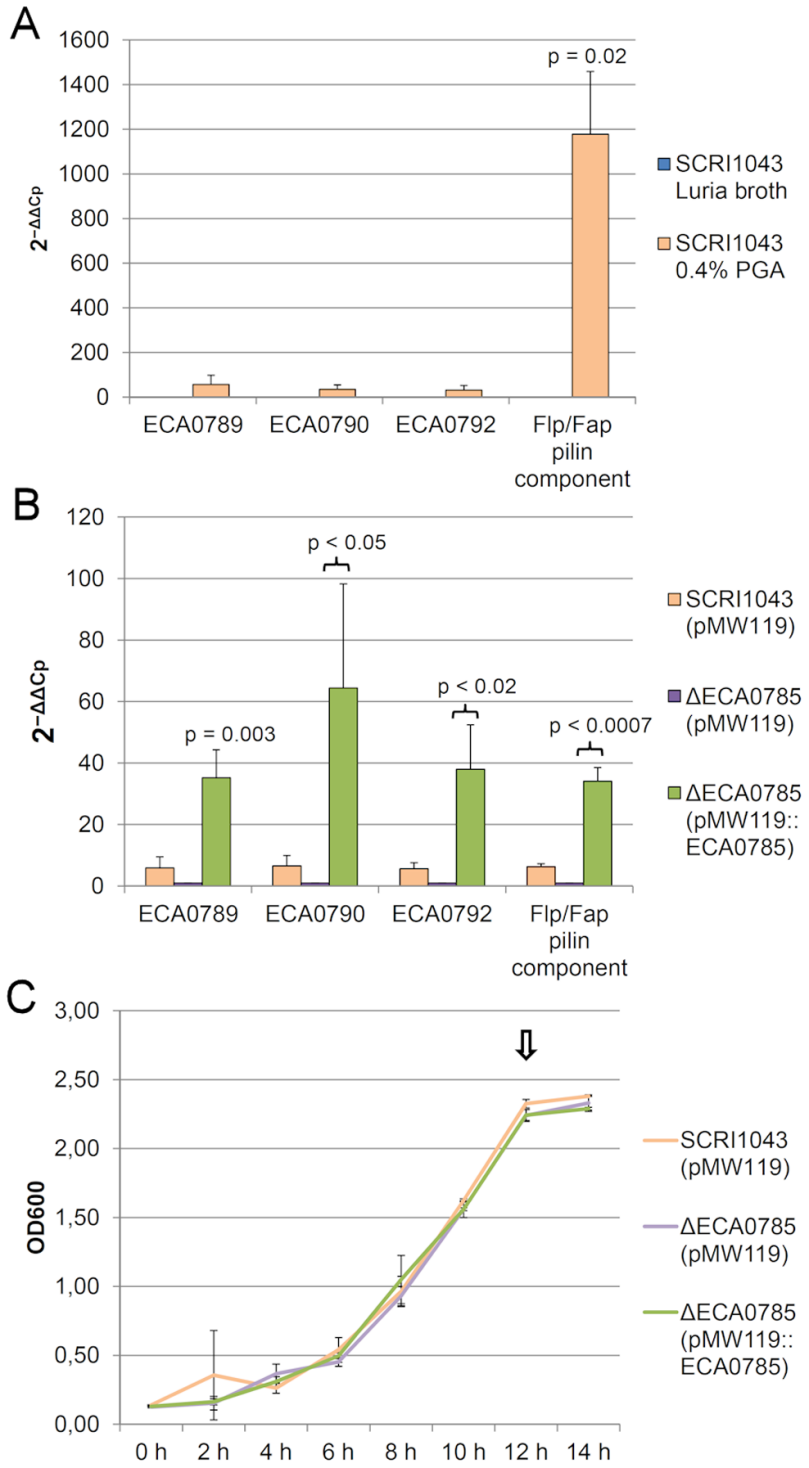
A novel two-component system in *Pectobacterium* regulates predicted Flp/Tad pilus-encoding genes. A) Genes encoding the Flp/Fap pilin component, RcpA (ECA0789), RcpB (ECA0790) and TadA (ECA0792) were upregulated under *in planta*-mimicking conditions (*hrp*-inducing minimal medium salts supplemented with 0.4% polygalacturonic acid = PGA) compared with their levels in rich medium (Luria broth) when measured by relative qPCR. However, despite relatively large fold changes, only the Flp/Fap pilin component result was statistically significant (p<0.02). Due to the analysis method used for the relative qPCR data (2^−ΔΔCt^), the values obtained from the Luria broth samples were normalized to be 1 and are thus very close to the x-axis on the left side of the column, which represents the relative fold change of PGA samples. B) A novel response regulator (ECA0785) affected the expression levels of genes encoding Flp/Fap pilin component, RcpA (ECA0789), RcpB (ECA0790) and TadA (ECA0792) (p<0.05) under conditions that induce *flp*/*tad* gene expression (*hrp*-inducing minimal medium salts supplemented with 0.4% polygalacturonic acid = PGA). The reduction of gene expression in the ΔECA0785 mutant was restored by *in trans* complementation (p<0.05). C) *In vitro* growth of *P. atrosepticum* SCRI1043 and its derivative ΔECA0785 in *hrp*-inducing minimal medium salts supplemented with 0.4% PGA at 28°C. The sampling point for the qPCR experiments is marked with an arrow. The experiments were repeated independently a minimum of three times, and the figures represent the averages and standard deviations of three independent experiments (A and B) or the averages of three replicates in a single experiment (C).

To explore the possibility that the corresponding TCS regulates the *flp*/*tad* gene cluster in soft-rot enterobacteria, we mutagenized the response regulator (ECA0785) in *P. atrosepticum* SCRI1043 and used relative qPCR to characterize the effect of this mutation on *flp*/*tad* gene expression in different growth media after the culture reached the stationary phase (Figure 3A, Figure 3B, Figure 3C), which corresponds the growth phase in where the *flp*/*tad* genes were originally characterized in *P. atrosepticum* SCRI1043 in our earlier work [10]. We examined the *flp*/*tad* genes that were differentially expressed in the VasH microarrays (Figure 2A: *rcpA*/ECA0789, *rcpB*/ECA0790 and *tadA*/ECA0792) and the Flp/Fap pilin component-encoding gene (start-stop; 861131–861352 in SCRI1043). The expression levels of these genes were increased under plant-mimicking conditions compared with rich growth medium (Luria broth) (Figure 3A). The expression levels of these *flp*/*tad* genes were significantly reduced in the ECA0785 response regulator mutant strain compared with the corresponding wild-type strain and were complemented *in trans*, suggesting that the TCS adjacent to the *flp*/*tad* gene cluster may be necessary for the expression of these genes (Figure 3B). These findings support the hypotheses that the Flp/Tad pilus encoding genes are expressed in *Pectobacterium*, the pilus is required under *in planta* conditions and the *flp*/*tad* gene cluster is, at least partially, regulated by the flanking novel two-component system in *Pectobacterium*.

### The Novel Response Regulator and Flp/Tad Pilus Genes are Both Necessary for Full Virulence of *Pectobacterium*

**Figure 4:**
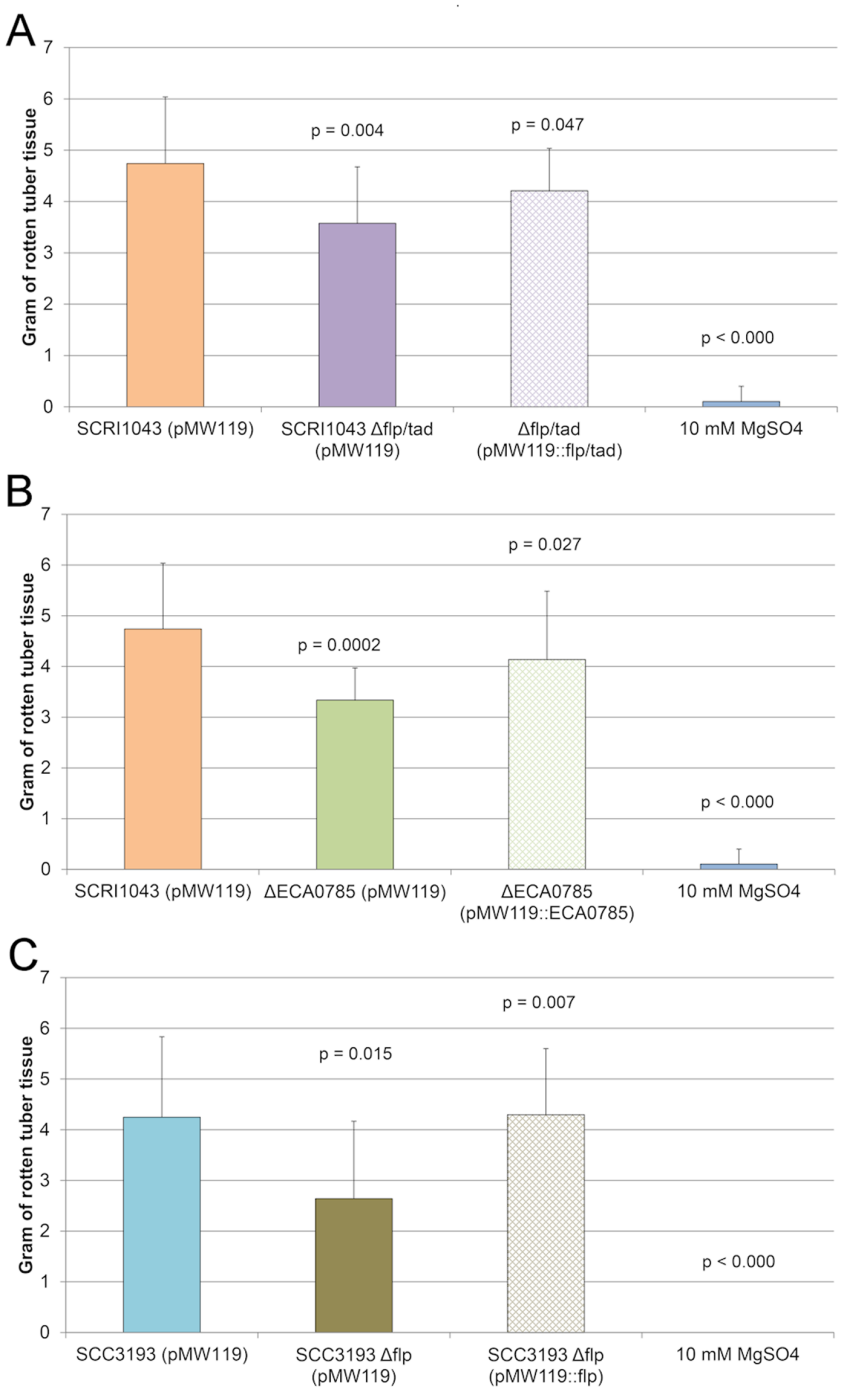
The predicted Flp/Tad pilus is necessary for full virulence of *Pectobacterium* in potato tubers. A) A mutant strain of *P. atrosepticum* SCRI1043 deficient in the expression of the full *flp*/*tad* gene cluster (Δ*flp*/*tad*) exhibited impaired maceration capacity in potato tubers compared with the wild-type strain (p = 0.004). The maceration capacity was complemented *in trans* (p = 0.047). B) A mutant strain of *P. atrosepticum* SCRI1043 deficient in the expression of a putative response regulator of the *flp*/*tad* gene cluster (ΔECA0785) also displayed impaired virulence in potato tubers (p = 0.0002), and the phenotype was restored by complementation *in trans* (p = 0.027). C) *P. wasabiae* SCC3193 lacking the predicted Flp/Fap pilin component-encoding gene (Δ*flp*) also displayed impaired maceration of potato tubers (p = 0.015), and the phenotype was complemented *in trans* (p = 0.007). As a negative control, 10 mM MgSO_4_ buffer was used, confirming that the symptoms are a consequence of the inoculated bacterial strains as opposed to the natural population of soft-rot bacteria. The virulence assays were repeated independently a minimum of three times, and the figures represent a single biological replicate (n = 10–15 tubers per strain).

To determine if the *flp*/*tad* gene cluster is necessary for full virulence of *Pectobacterium* in potato tubers, *rcpA*, *rcpB*, *tadZ* and *tadA* (ECA0789–ECA0792, respectively) were deleted as a cluster from *P. atrosepticum* SCRI1043, and W5S_0783 (Flp/Fap pilin component) was deleted from *P. wasabiae* SCC3193. In both *P. atrosepticum* and *P. wasabiae*, these regions were replaced with an antibiotic cassette. The resulting mutants could be complemented *in trans* by introducing the corresponding wild-type alleles into the mutants. Bacteria (∼10^7^ cfu) were inoculated into potato tubers and incubated under conditions favoring the development of soft-rot symptoms. After incubation, the softened tuber tissue was weighed, and the results were analyzed statistically. In this model, the *P. atrosepticum* SCRI1043 *flp*/*tad* partial cluster mutant (Δ*flp*/*tad*), the *P. atrosepticum* SCRI1043 regulator mutant (ΔECA0785) and the *P. wasabiae* SCC3193 Flp/Fap pilin component mutant (Δ*flp*) displayed a significant delay in symptoms compared with the wild-type strains and the respective mutant strains complemented *in trans* (Figure 4A, Figure 4B, Figure 4C).

**Figure 5:**
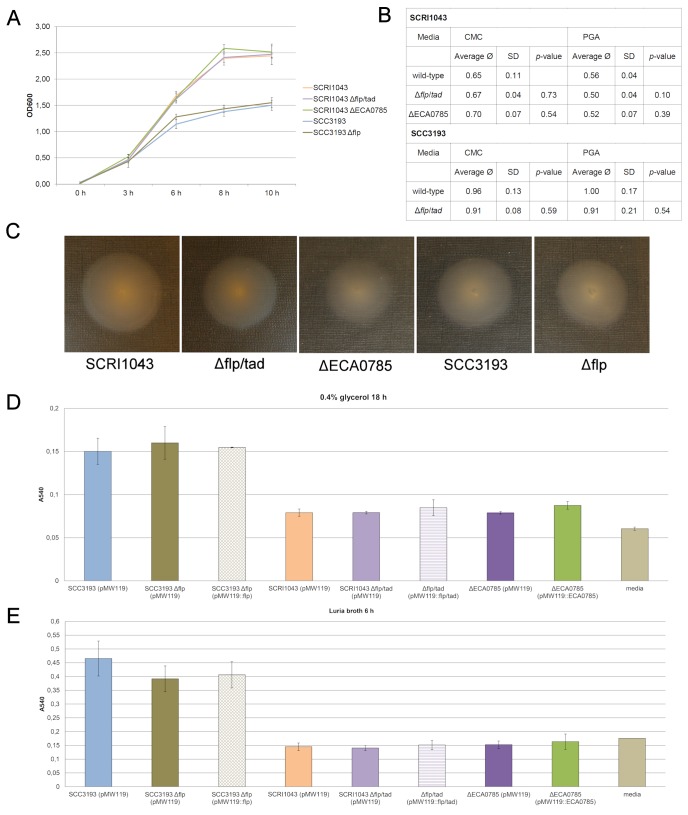
The predicted Flp/Tad pilus has no effect on growth, PCWDEs, motility or biofilm formation *in vitro*. A) *In vitro* growth of *P. atrosepticum* SCRI1043, *P. wasabiae* SCC3193 and their derivatives (Δ*flp*/*tad*, ΔECA0785 and Δ*flp*) in *hrp*-inducing minimal media supplemented with 10% v/v potato tuber extract. *P. wasabiae* and *P. atrosepticum* reached different cell densities, although there was no significant difference between the wild-type strains and the corresponding mutant strains. B) Production of plant cell wall-degrading enzymes (PCWDEs) in *P. atrosepticum* SCRI1043, *P. wasabiae* SCC3193 and their derivatives (Δ*flp*/*tad*, ΔECA0785 and Δ*flp*) growing on indicator plates containing 0.7% polygalacturonic acid (PGA) or 0.5% carboxymethylcellulose (CMC). In the figure, “average Ø” indicates the diameter of the halo around the bacteria in centimeters. The averages and standard deviations (SD) of four replicates (n = 4) are provided, and the experiment was repeated a minimum of three times with similar results. C) Flagella-based motility of *P. atrosepticum* SCRI1043, *P. wasabiae* SCC3193 and their derivatives (Δ*flp*/*tad*, ΔECA0785 and Δ*flp*) on 0.25% agar plates. All strains were motile, and no significant differences in spreading were observed. D) The *in vitro* biofilm formation ability of *P. atrosepticum* SCRI1043 and *P. wasabiae* SCC3193 differed in 0.4% glycerol after 18 h of incubation, but there was no significant difference between the wild-type strains and the corresponding mutant strains (Δ*flp*/*tad*, ΔECA0785, Δ*flp*). E) The *in vitro* biofilm formation of *P. atrosepticum* SCRI1043 and *P. wasabiae* SCC3193 differed in Luria broth after 6 h of incubation, but there was no difference between the wild-type strains and the corresponding mutant strains (Δ*flp*/*tad*, ΔECA0785 and Δ*flp*). All experiments were repeated a minimum of three times with a minimum of three replicates. The figures represent the averages and standard deviations of one experiment.

Based on current knowledge, the virulence determinants of soft-rot enterobacteria are either connected via a multilevel regulatory network and/or are induced under similar conditions in response to environmental stimuli. It is also possible that the mutagenized strains carry secondary mutations that affect virulence determinants, although the *in trans* complementation suggests that the altered phenotype is indeed a consequence of the deletion or inactivation of the gene of interest. We experimentally clarified whether the inactivation of *flp*/*tad* genes or the response regulator has an effect on other major virulence determinants or on basic metabolic functions such as population growth, the production of PCWDEs and flagella-based motility. Under the experimental conditions employed in this study, no significant differences were observed between the mutant strains (Δ*flp*/*tad*, ΔECA0785 and Δ*flp*) and the corresponding wild-type strains (SCRI1043 or SCC3193) (Figure 5A, Figure 5B, Figure 5C). Under growth conditions mimicking *in planta* conditions, *P. atrosepticum* reached a higher cell density than *P. wasabiae*; however, there was no difference between the mutagenized strains and the wild-type strain (Figure 5A). In assays measuring PCWDE (pectinases and cellulases) production, *P. wasabiae* performed better than *P. atrosepticum*, although there were no differences between the mutagenized strains and the wild-type strain (Figure 5B). In the flagella-based motility assays, all tested strains were motile, and no significant differences in the dispersion speed were observed among the bacterial strains (Figure 5C). Based on these experiments, it is plausible that the altered maceration ability of the response regulator mutant (ΔECA0785) or the *flp*/*tad* mutants (Δ*flp*/*tad* and Δ*flp*) is not a consequence of alterations in other major virulence determinants. Although we cannot rule out the possibility that the response regulator has targets other than the *flp*/*tad* gene cluster, owing to the similarly reduced virulence phenotype of the *flp*/*tad* gene cluster mutants (Figure 4A, Figure 4B), we suggest that the impact of this novel virulence regulator is mediated mainly through the regulation of the *flp*/*tad* gene cluster.

In light of the role of the Flp/Tad pilus in biofilm formation in other bacteria [32], [33], [34], [17], we investigated whether the *flp*/*tad* gene cluster plays a role in biofilm formation in *Pectobacterium*. In biofilm formation assays, *P. wasabiae* SCC3193 attached well into the polypropylene surface independent of the culture medium, but *P. atrosepticum* SCRI1043 generated little or no film under the same conditions (Figure 5D, Figure 5E). However, none of the mutagenized strains (Δ*flp*/*tad*, Δ*flp* and ΔECA0785) exhibited differential phenotypes when compared to the wild-type strain in the biofilm formation assays (Figure 5D, Figure 5E). Therefore, the function of the Flp/Tad pilus in biofilm formation in *Pectobacterium* remains unknown. It is possible that pilus formation is regulated both transcriptionally and post-transcriptionally and that the pilus is generated only under the right conditions such as when the bacterium is in contact with plant tissue.

## Discussion

In the current study, our aim was to examine the expression, regulation and role in virulence of the predicted Flp/Tad pilus in soft-rot enterobacteria of the genus *Pectobacterium*. The Flp/Tad pilus is a well characterized and visualized bacterial surface structure that has been observed in several animal pathogenic bacteria, an environmental bacterium, a human gut bacterium and in the phytopathogen *R. solanacearum* [11], [16], [17]. We previously demonstrated that *flp*/*tad* genes are upregulated in potato tubers mimicking *in vitro* conditions (apoplast vs. tuber vs. stem), along with T6SS genes [10], indicating that the Flp/Tad pilus may be co-regulated with T6SS and is essential for soft-rot enterobacteria *in planta*.

Comparative genomics analysis indicated that the *flp*/*tad* gene cluster is present in the genus *Pectobacterium* and in the strain *Dickeya zeae* Ech1591 but is otherwise absent in the genus *Dickeya*. The synteny of the cluster is highly similar to that of previously characterized clusters in several animal pathogens (Figure 1). The Flp/Tad pilus cluster has often been referred to as a Widespread Colonization Island, which is a target of horizontal gene transfer [35], [11]. Horizontal gene transfer enables the acquisition of beneficial traits from distantly related species [36]. The presence of a similar *flp*/*tad* gene cluster in distantly related bacterial species and the lack of the cluster in some closely related soft-rot enterobacteria together with data from previous studies [35], [11] indicate that the *flp*/*tad* gene cluster may have a horizontal origin in soft-rot enterobacteria. It is also likely that the cluster encodes the Flp/Tad pilus because a similar cluster encodes Flp/Tad pilus in the well-studied bacterial species *A. actinomycetemcomitans* [32], [14], [37], [38], [39], [40].

Three *flp*/*tad* cluster genes were identified in our microarray data (Figure 2). When comparing the *P. atrosepticum* SCRI1043 wild-type strain and its derivative Δ*vasH* (VasH is a sigma54-dependent transcriptional activator), none of these *flp*/*tad* genes were differentially expressed or complemented as analyzed by relative qPCR, which is a more sensitive technique than microarray analysis (Figure 2). T6SS and the Flp/Tad pilus are likely required under similar environmental conditions in soft-rot enterobacteria, and they may be induced, at least partially, by the same external stimuli for example, plant tissue degradation products as noted earlier [10]); however, we were unable to confirm actual cross-talk between these two systems, at least not via VasH. Two major global regulators of virulence (the quorum sensing synthetase ExpI and the virulence gene repressor RsmA) that affect global gene expression on the transcriptional level were previously investigated using microarray techniques [41], [9]. Flp/Tad pilus-related genes were not present in the microarray data in either of these studies, indicating that the *flp*/*tad* gene cluster is not controlled by quorum sensing or RsmA regulation in *Pectobacterium*. This is in contrast to most other characterized virulence determinants, such as PCWDEs, the type I secretion system (T1SS), the type II secretion system (T2SS), T3SS and T6SS (quorum sensing [41]) and PCWDEs, T6SS, Flagella and LPS synthesis (RsmA [9]). Our work, together with previous studies, suggests that the Flp/Tad pilus in *Pectobacterium* may be regulated by a previously unknown regulatory system.

Based on comparative genomics (Figure 1), we identified a putative TCS adjacent to the *flp*/*tad* gene cluster in the soft-rot enterobacteria, *Brenneria* and *Marinomonas*. Mutagenesis of the response regulator and *in trans* complementation of the mutant had significant effects on the expression of *flp*/*tad* genes in *P. atrosepticum* SCRI1043 (Figure 3), indicating that we have discovered a regulator for the *flp*/*tad* gene cluster and hence a novel regulator in *Pectobacterium*. The *flp*/*tad* gene cluster and the flanking TCS are predicted to be transcribed in 5 operons in *P. wasabiae* SCC3193, similar to corresponding genes in *P. atrosepticum* SCRI1043 [6]. The regulation of the *flp*/*tad* genes has been investigated in several animal pathogens [38], [39], [42], [15], but a regulatory system similar to that which we have characterized in *Pectobacterium* has not been reported. In *Pseudomonas aeruginosa*, the *flp*/*tad* gene cluster is regulated by another TCS [15], and there is no significant sequence similarity to the TCS of *Pectobacterium* (in a blastx comparison of the Flp/Fap pilin component of *P. atrosepticum* SCRI1043 and *P. aeruginosa* PAO1, no significant similarity was found). Based on this study and previous studies, we suggest that the TCS described in this work is a novel regulator and that it regulates the *flp*/*tad* gene cluster in *Pectobacterium* and, potentially, that in *Brenneria* and *Marinomonas*.

To characterize the role of the Flp/Tad pilus in virulence of *Pectobacterium*, we performed virulence assays in potato tubers. In these virulence assays, we compared the maceration efficiency of the mutant strains (lacking *flp*/*tad* genes or the novel Flp/Tad pilus response regulator) with that of the wild-type strain and the *in trans*-complemented mutant strains. Initially, we performed several preliminary virulence assays to determine the best conditions for these mutants. Unexpectedly, neither the *flp*/*tad* mutant strains nor the TCS regulatory mutant were as effective in disease development as the wild-type strain, even though a significant number of cells (10^7^ cfu per inoculation site) were inoculated (Figure 4). The Flp/Tad pilus has been shown to be necessary for attachment and biofilm formation [32], [33], [34], [17]; thus, it was anticipated that the pilus in soft-rot enterobacteria would be required for attachment to the plant tissue and might have a greater impact during the initial steps of the infection. The delayed disease development and *in planta* complementation of the *flp*/*tad* mutant strains compared with the wild-type strain in this study (Figure 4) could have occurred because the *flp*/*tad* gene cluster in soft-rot enterobacteria is necessary for the organization of the bacterial population into a biofilm-like structure during the maceration stage; however, no direct evidence is available to support this hypothesis. Our biofilm assays on abiotic surfaces (Figure 5) did not support the hypothesis that the Flp/Tad pilus plays a role in attachment to surfaces or in biofilm formation. Furthermore, in the case of *P. atrosepticum*, it was unclear if the cells were attached to the abiotic surface. Overall, *P. atrosepticum* rarely forms a biofilm-like structure under abiotic conditions, with the exception of LPS-deficient mutant strains of *P. atrosepticum* SCRI1043 [7] and the c-di-GMP-overexpressing *P. atrosepticum* SCRI1043 [43], which produce more biofilm than the wild-type strain. However, in *Haemophilus ducreyi*, the *flp*/*tad* gene cluster is required for attachment to a plastic surface and to HFF cells as well as for microcolony formation when co-cultured with HFF cells; however, it has no effect on virulence in animal models [33], suggesting a specific role for this gene cluster in bacterial population structure during biofilm formation rather than a role in attachment to the host tissue during infection. It is also possible that the *flp*/*tad* gene cluster in *Pectobacterium* is tightly regulated *in planta*, and our *in vitro* assays did not support pilus formation. In conclusion, we suggest that the *flp*/*tad* gene cluster in *Pectobacterium* is a novel virulence determinant and that its mechanistic function provides an interesting target for future investigations.

Despite the unknown virulence mechanism of the *flp*/*tad* gene cluster in soft-rot enterobacteria, we suggest that the cluster consists of genes encoding the Flp/Tad pilus and an adjacent transcriptional activator (a response regulator in the TCS adjacent to the *flp*/*tad* gene cluster). It is plausible that the observed decrease in the virulence of *flp*/*tad* mutants and their *in trans* complementation with the wild-type alleles is due to their specific function in Flp/Tad pilus regulation *in planta* rather than due to a secondary mutation or signaling related to other known virulence determinants. To our knowledge, no pilus (secretion of its own structural proteins only) or fimbria has been characterized as having a significant effect on the virulence of soft-rot enterobacteria. The response regulator may be a novel regulator in soft-rot enterobacteria and may be one of a few specific regulators that function independently of the regulatory network to solely regulate the biogenesis of the Flp/Tad pilus, which has a significant effect on the virulence of *Pectobacterium* in potato tubers.

## Supporting Information

Figure S1 **Alignment of predicted Flp/Fap pilin component-encoding genes of** ***Pectobacterium*** **.**
(DOC)

Table S1 Bacterial strains, plasmids and primers utilized in this study.
(DOC)

## References

[1] Ma B, Hibbing ME, Kim H-S, Reedy RM, Yedidia I, et al (2007) Host range and molecular phylogenies of the soft rot enterobacterial genera pectobacterium and dickeya. Phytopathology 97: 1150–1163 doi:10.1094/PHYTO-97-9-1150 18944180 10.1094/PHYTO-97-9-1150

[2] Pitman AR, Harrow SA, Visnovsky SB (2009) Genetic characterisation of Pectobacterium wasabiae causing soft rot disease of potato in New Zealand. Eur J Plant Pathol 126: 423–435 doi:10.1007/s10658-009-9551-y

[3] Czajkowski R, Pérombelon MCM, van Veen JA, van der Wolf JM (15:04:53) Control of blackleg and tuber soft rot of potato caused by Pectobacterium and Dickeya species: a review. Plant Pathol. Available: http://onlinelibrary.wiley.com/doi/10.1111/j.1365-3059.2011.02470.x/abstract. Accessed 11 October 2011.

[4] Toth IK, van der Wolf JM, Saddler G, Lojkowska E, Hélias V, et al (2011) Dickeya species: an emerging problem for potato production in Europe. Plant Pathol 60: 385–399 doi:–10.1111/j.1365–3059.2011.02427.x

[5] Charkowski A, Blanco C, Condemine G, Expert D, Franza T, et al (2012) The role of secretion systems and small molecules in soft-rot enterobacteriaceae pathogenicity. Annu Rev Phytopathol 50: 425–449 doi:10.1146/annurev-phyto-081211-173013 22702350 10.1146/annurev-phyto-081211-173013

[6] Nykyri J, Niemi O, Koskinen P, Nokso-Koivisto J, Pasanen M, et al (2012) Revised phylogeny and novel horizontally acquired virulence determinants of the model soft rot phytopathogen Pectobacterium wasabiae SCC3193. Plos Pathog 8: e1003013 doi:10.1371/journal.ppat.1003013 23133391 10.1371/journal.ppat.1003013 PMC3486870

[7] Evans TJ, Ind A, Komitopoulou E, Salmond GPC (2010) Phage-selected lipopolysaccharide mutants of Pectobacterium atrosepticum exhibit different impacts on virulence. J Appl Microbiol 109: 505–514 doi:–10.1111/j.1365–2672.2010.04669.x 20132374 10.1111/j.1365-2672.2010.04669.x

[8] Põllumaa L, Alamäe T, Mäe A (2012) Quorum sensing and expression of virulence in pectobacteria. Sensors 12: 3327–3349 doi:10.3390/s120303327 22737011 10.3390/s120303327 PMC3376562

[9] Kõiv V, Andresen L, Broberg M, Frolova J, Somervuo P, et al (2013) Lack of RsmA-mediated control results in constant hypervirulence, cell elongation, and hyperflagellation in Pectobacterium wasabiae. Plos One 8: e54248 doi:10.1371/journal.pone.0054248 23372695 10.1371/journal.pone.0054248 PMC3553148

[10] Mattinen L, Somervuo P, Nykyri J, Nissinen R, Kouvonen P, et al (2008) Microarray profiling of host-extract-induced genes and characterization of the type VI secretion cluster in the potato pathogen Pectobacterium atrosepticum. Microbiol Read Engl 154: 2387–2396 doi:10.1099/mic.0.2008/017582-0 10.1099/mic.0.2008/017582-0 18667571

[11] Tomich M, Planet PJ, Figurski DH (2007) The tad locus: postcards from the widespread colonization island. Nat Rev Microbiol 5: 363–375 doi:10.1038/nrmicro1636 17435791 10.1038/nrmicro1636

[12] Giltner CL, Nguyen Y, Burrows LL (2012) Type IV pilin proteins: versatile molecular modules. Microbiol Mol Biol Rev Mmbr 76: 740–772 doi:10.1128/MMBR.00035-12 23204365 10.1128/MMBR.00035-12 PMC3510520

[13] Perez-Cheeks BA, Planet PJ, Sarkar IN, Clock SA, Xu Q, et al (2012) The product of tadZ, a new member of the parA/minD superfamily, localizes to a pole in Aggregatibacter actinomycetemcomitans. Mol Microbiol 83: 694–711 doi:–10.1111/j.1365–2958.2011.07955.x 22239271 10.1111/j.1365-2958.2011.07955.x PMC3305808

[14] Bhattacharjee MK, Kachlany SC, Fine DH, Figurski DH (2001) Nonspecific adherence and fibril biogenesis by Actinobacillus actinomycetemcomitans: TadA protein is an ATPase. J Bacteriol 183: 5927–5936 doi:10.1128/JB.183.20.5927-5936.2001 11566992 10.1128/JB.183.20.5927-5936.2001 PMC99671

[15] Bernard CS, Bordi C, Termine E, Filloux A, de Bentzmann S (2009) Organization and PprB-dependent control of the Pseudomonas aeruginosa tad Locus, involved in Flp pilus biology. J Bacteriol 191: 1961–1973 doi:10.1128/JB.01330-08 19151143 10.1128/JB.01330-08 PMC2648354

[16] O’Connell Motherway M, Zomer A, Leahy SC, Reunanen J, Bottacini F, et al (2011) Functional genome analysis of Bifidobacterium breve UCC2003 reveals type IVb tight adherence (Tad) pili as an essential and conserved host-colonization factor. Proc Natl Acad Sci U S A 108: 11217–11222 doi:10.1073/pnas.1105380108 21690406 10.1073/pnas.1105380108 PMC3131351

[17] Wairuri CK, van der Waals JE, van Schalkwyk A, Theron J (2012) Ralstonia solanacearum needs Flp pili for virulence on potato. Mol Plant-Microbe Interactions Mpmi 25: 546–556 doi:10.1094/MPMI-06-11-0166 10.1094/MPMI-06-11-0166 22168446

[18] Hinton JC, Perombelon MC, Salmond GP (1985) Efficient transformation of Erwinia carotovora subsp. carotovora and E. carotovora subsp. atroseptica. J Bacteriol 161: 786–788. 3968041 10.1128/jb.161.2.786-788.1985 PMC214956

[19] Pirhonen M, Heino P, Helander I, Harju P, Palva ET (1988) Bacteriophage T4 resistant mutants of the plant pathogen Erwinia carotovora. Microb Pathog 4: 359–367. 3241545 10.1016/0882-4010(88)90063-0

[20] Altschul SF, Madden TL, Schäffer AA, Zhang J, Zhang Z, et al (1997) Gapped BLAST and PSI-BLAST: a new generation of protein database search programs. Nucleic Acids Res 25: 3389–3402. 9254694 10.1093/nar/25.17.3389 PMC146917

[21] Altschul SF, Wootton JC, Gertz EM, Agarwala R, Morgulis A, et al (2005) Protein database searches using compositionally adjusted substitution matrices. Febs J 272: 5101–5109 doi:10.1111/j.1742-4658.2005.04945.x 16218944 10.1111/j.1742-4658.2005.04945.x PMC1343503

[22] Datsenko KA, Wanner BL (2000) One-step inactivation of chromosomal genes in Escherichia coli K-12 using PCR products. Proc Natl Acad Sci U S A 97: 6640–6645 doi:10.1073/pnas.120163297 10829079 10.1073/pnas.120163297 PMC18686

[23] Summers WC (1970) A simple method for extraction of RNA from E. coli utilizing diethyl pyrocarbonate. Anal Biochem 33: 459–463. 4910776 10.1016/0003-2697(70)90316-7

[24] Takle GW, Toth IK, Brurberg MB (2007) Evaluation of reference genes for real-time RT-PCR expression studies in the plant pathogen Pectobacterium atrosepticum. Bmc Plant Biol 7: 50 doi:10.1186/1471-2229-7-50 17888160 10.1186/1471-2229-7-50 PMC2151947

[25] Livak KJ, Schmittgen TD (2001) Analysis of relative gene expression data using real-time quantitative PCR and the 2(-Delta Delta C(T)) Method. Methods San Diego Calif 25: 402–408 doi:10.1006/meth.2001.1262 10.1006/meth.2001.1262 11846609

[26] Edgar R, Domrachev M, Lash AE (2002) Gene Expression Omnibus: NCBI gene expression and hybridization array data repository. Nucleic Acids Res 30: 207–210 doi:10.1093/nar/30.1.207 11752295 10.1093/nar/30.1.207 PMC99122

[27] Pirhonen M, Flego D, Heikinheimo R, Palva ET (1993) A small diffusible signal molecule is responsible for the global control of virulence and exoenzyme production in the plant pathogen Erwinia carotovora. Embo J 12: 2467–2476. 8508772 10.1002/j.1460-2075.1993.tb05901.x PMC413482

[28] O’Toole GA, Kolter R (1998) Flagellar and twitching motility are necessary for Pseudomonas aeruginosa biofilm development. Mol Microbiol 30: 295–304. 9791175 10.1046/j.1365-2958.1998.01062.x

[29] Bell KS, Sebaihia M, Pritchard L, Holden MTG, Hyman LJ, et al (2004) Genome sequence of the enterobacterial phytopathogen Erwinia carotovora subsp. atroseptica and characterization of virulence factors. Proc Natl Acad Sci U S A 101: 11105–11110 doi:10.1073/pnas.0402424101 15263089 10.1073/pnas.0402424101 PMC503747

[30] Bernard CS, Brunet YR, Gavioli M, Lloubès R, Cascales E (2011) Regulation of type VI secretion gene clusters by sigma54 and cognate enhancer binding proteins. J Bacteriol 193: 2158–2167 doi:10.1128/JB.00029-11 21378190 10.1128/JB.00029-11 PMC3133059

[31] Kitaoka M, Miyata ST, Brooks TM, Unterweger D, Pukatzki S (2011) VasH is a transcriptional regulator of the type VI secretion system functional in endemic and pandemic Vibrio cholerae. J Bacteriol 193: 6471–6482 doi:10.1128/JB.05414-11 21949076 10.1128/JB.05414-11 PMC3232897

[32] Kachlany SC, Planet PJ, Desalle R, Fine DH, Figurski DH, et al (2001) flp-1, the first representative of a new pilin gene subfamily, is required for non-specific adherence of Actinobacillus actinomycetemcomitans. Mol Microbiol 40: 542–554. 11359562 10.1046/j.1365-2958.2001.02422.x

[33] Nika JR, Latimer JL, Ward CK, Blick RJ, Wagner NJ, et al (2002) Haemophilus ducreyi requires the flp gene cluster for microcolony formation in vitro. Infect Immun 70: 2965–2975. 12010986 10.1128/IAI.70.6.2965-2975.2002 PMC127968

[34] De Bentzmann S, Aurouze M, Ball G, Filloux A (2006) FppA, a novel Pseudomonas aeruginosa prepilin peptidase involved in assembly of type IVb pili. J Bacteriol 188: 4851–4860 doi:10.1128/JB.00345-06 16788194 10.1128/JB.00345-06 PMC1483019

[35] Planet PJ, Kachlany SC, Fine DH, DeSalle R, Figurski DH (2003) The Widespread Colonization Island of Actinobacillus actinomycetemcomitans. Nat Genet 34: 193–198 doi:10.1038/ng1154 12717435 10.1038/ng1154

[36] Juhas M, van der MeerJR, Gaillard M, Harding RM, Hood DW, et al (2009) Genomic islands: tools of bacterial horizontal gene transfer and evolution. Fems Microbiol Rev 33: 376–393 doi:10.1111/j.1574-6976.2008.00136.x 19178566 10.1111/j.1574-6976.2008.00136.x PMC2704930

[37] Tomich M, Fine DH, Figurski DH (2006) The TadV protein of Actinobacillus actinomycetemcomitans is a novel aspartic acid prepilin peptidase required for maturation of the Flp1 pilin and TadE and TadF pseudopilins. J Bacteriol 188: 6899–6914 doi:10.1128/JB.00690-06 16980493 10.1128/JB.00690-06 PMC1595517

[38] Perez BA, Planet PJ, Kachlany SC, Tomich M, Fine DH, et al (2006) Genetic analysis of the requirement for flp-2, tadV, and rcpB in Actinobacillus actinomycetemcomitans biofilm formation. J Bacteriol 188: 6361–6375 doi:10.1128/JB.00496-06 16923904 10.1128/JB.00496-06 PMC1595400

[39] Kram KE, Hovel-Miner GA, Tomich M, Figurski DH (2008) Transcriptional regulation of the tad locus in Aggregatibacter actinomycetemcomitans: a termination cascade. J Bacteriol 190: 3859–3868 doi:10.1128/JB.00128-08 18375561 10.1128/JB.00128-08 PMC2395055

[40] Clock SA, Planet PJ, Perez BA, Figurski DH (2008) Outer membrane components of the Tad (tight adherence) secreton of Aggregatibacter actinomycetemcomitans. J Bacteriol 190: 980–990 doi:10.1128/JB.01347-07 18055598 10.1128/JB.01347-07 PMC2223556

[41] Liu H, Coulthurst SJ, Pritchard L, Hedley PE, Ravensdale M, et al (2008) Quorum sensing coordinates brute force and stealth modes of infection in the plant pathogen Pectobacterium atrosepticum. Plos Pathog 4: e1000093 doi:10.1371/journal.ppat.1000093 18566662 10.1371/journal.ppat.1000093 PMC2413422

[42] Schilling J, Wagner K, Seekircher S, Greune L, Humberg V, et al (2010) Transcriptional activation of the tad type IVb pilus operon by PypB in Yersinia enterocolitica. J Bacteriol 192: 3809–3821 doi:10.1128/JB.01672-09 20472801 10.1128/JB.01672-09 PMC2897337

[43] Pérez-Mendoza D, Coulthurst SJ, Sanjuán J, Salmond GPC (2011) N-Acetylglucosamine-dependent biofilm formation in Pectobacterium atrosepticum is cryptic and activated by elevated c-di-GMP levels. Microbiol Read Engl 157: 3340–3348 doi:10.1099/mic.0.050450-0 10.1099/mic.0.050450-0 21948048

