# Supplementary material for: Role and Regulation of the Flp/Tad Pilus in the Virulence of Pectobacterium atrosepticum SCRI1043 and Pectobacterium wasabiae SCC3193
Source: PLoS One. 2013 Sep 9;8(9):e73718. doi: 10.1371/journal.pone.0073718 (PMC3767616; doi:10.1371/journal.pone.0073718)
Supplement: Figure S1 — Alignment of predicted Flp/Fap pilin component-encoding genes of Pectobacterium . (DOC) [file pone.0073718.s001.doc]

**
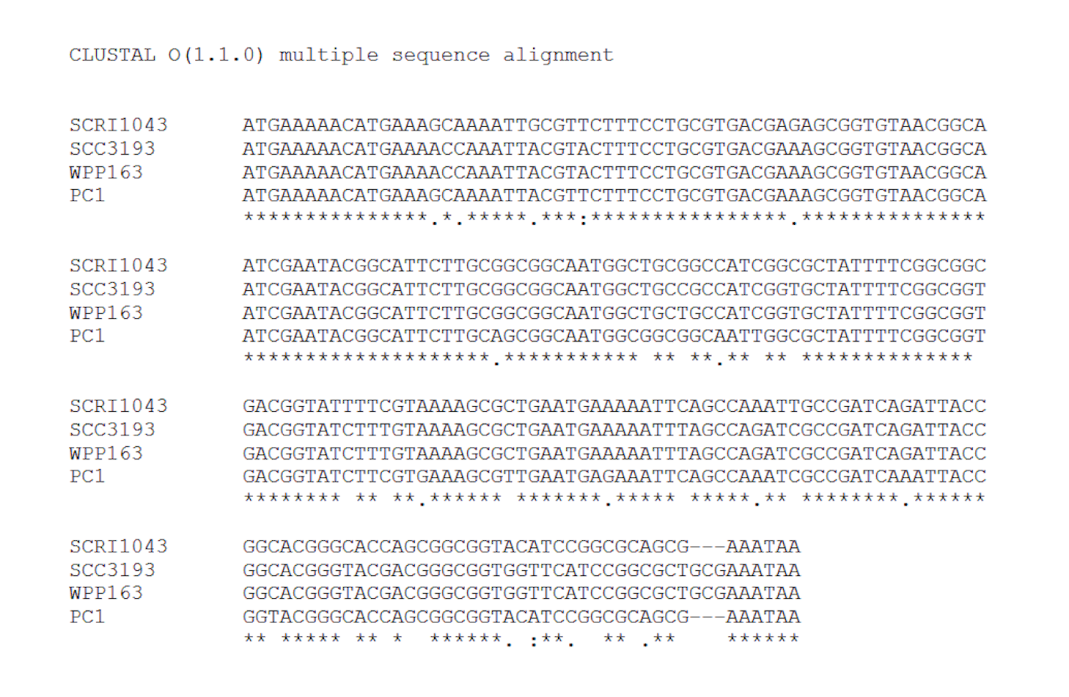
**

**Figure S1. Alignment of predicted Flp/Fap pilin component-encoding genes of *Pectobacterium*.** The ORF of a putative Flp/Fap pilin component (fimbrial low-molecular-weight protein/fibril-associated protein) of *P. atrosepticum* SCRI1043 was characterized. An alignment of this ORF with other Flp/Fap pilin component ORFs in selected *Pectobacterium* species (*P. wasabiae* SCC3193, *P. wasabiae* WPP163 and *P. aroidearum* PC1) is shown. The Flp/Fap pilin component protein is a single unit of the Flp/Tad pilin structure. The ORF alignment is based on sequences obtained from the public database in NCBI, and the alignment was performed using Clustal Omega.
