# Supplementary material for: Role and Regulation of the Flp/Tad Pilus in the Virulence of Pectobacterium atrosepticum SCRI1043 and Pectobacterium wasabiae SCC3193
Source: PLoS One. 2013 Sep 9;8(9):e73718. doi: 10.1371/journal.pone.0073718 (PMC3767616; doi:10.1371/journal.pone.0073718)
Supplement: Table S1 — Bacterial strains, plasmids and primers utilized in this study. (DOC) [file pone.0073718.s002.doc]

**Table S**1. Strains, plasmids and primers utilized in the study.

| **Strains** | **Descriptions** | **References** |
| --- | --- | --- |
| *Pectobacterium atrosepticum* SCRI1043 | wild-type strain | Hinton et al, 1985 |
| *Pectobacterium wasabiae* SCC3193 | wild-type strain | Pirhonen et al, 1988 |
| *P. atrosepticum* Δ*flp*/*tad* | A cluster mutant: ECA0788-ECA0793::Cm^R^ in SCRI1043 background | This study |
| *P. atrosepticum* ΔECA0785 | ECA0785::Cm^R^ in SCRI1043 background | This study |
| *P. atrosepticum* Δ*vasH* | ECA3435::Cm^R^ in SCRI1043 background | Mattinen et al, 2008 |
| *P. wasabiae* Δ*flp* | W5S_0783::Cm^R^ in SCC3193 background | This study |
| *E. coli* DH5α | For cloning | Laboratory strain |
| **Plasmids** | **Descriptions** | **References** |
| pKD46 | λRED expressing helping plasmid, Amp^R^ | Datsenko and Wanner, 2000 |
| pKD3 | Template plasmid for Cm^R^ cassette | Datsenko and Wanner, 2000 |
| pMW119 | Transcription vector, Amp^R^ | Nippon gene Co. Japan |
| pMW119::*flp*/*tad* | ECA0788-ECA0793 BamHI-SacI cloned into pMW119 | This study |
| pMW119::ECA0785 | ECA0785 BamHI-SacI cloned into pMW119 | This study |
| pMW119::*vasH* | ECA3435 HindIII-SacI cloned into pMW119 | This study |
| pMW119::*flp* | ECA0788-ECA0793 BamHI-SacI cloned into pMW119 | This study |
| **Primers for mutagenizations** | **Sequences** | **References** |
| P1_Δ*flp*/*tad* | ggtgccgcgagttgctcatagaacaatgaagcccattacg  gaaattaagtGTGTAGGCTGGAGCTGCTTC | This study |
| P2_Δ*flp*/*tad* | ctagcgccaacagcagtaagggatcgtcaaaaacagtca  tgagtggctctATGGGAATTAGCCATGGTCC | This study |
| P1_ECA0785 | cattaaccctgaattaattaggtaataagccacgtttatagg  gggaaattGTGTAGGCTGGAGCTGCTTC | This study |
| P2_ECA0785 | agtgttttatttttatctgccagcgctgccccagcttctttattt  ttattCATATGAATATCCTCCTTAG | This study |
| Flp_pilin_P1 (Δ*flp*) | gaattccttgtaggccatgatttctttggcggcggataga gaatctttccGTGTAGGCTGGAGCTGCTTC | This study |
| Flp_pilin_P2 (Δ*flp*) | tcagcagtgccgtctgcagccagtgcgtgtaatccgccat tttgcgttaaCATATGAATATCCTCCTTAG | This study |
| **Confirmation primers for mutagenizations** | **Sequences** | **References** |
| C1 | ttatacgcaaggcgacaagg | Datsenko and Wanner, 2000 |
| C2 | gatcttccgtcacaggtagg | Datsenko and Wanner, 2000 |
| Δ*flp*/*tad*_R (+C2) | ccgaggtcgaatacacttcc | This study |
| Δ*flp*/*tad*_fl_Fw | cgtcctacaacctatcaaaccg | This study |
| Δ*flp*/*tad*_fl_Rv | ctatagaggcgctgggtaaatg | This study |
| ECA0785_fl_Fw | cgcctaacctaagaactgtgtc | This study |
| ECA0785_fl_Rv | ggtaattcatcacccttcgctc | This study |
| Flp_pilin_fl._Fw | aaccgcagttagtccgtcta | This study |
| Flp_pilin_fl._Rv | gcggttaccatgatgaaaac | This study |
| **Primers for complementations** | **Sequences** | **References** |
| OPFlpBamHI | gacaattggatccctggcaaccgatatctacggggcatactca | This study |
| OPFlpSacI | ttataaaagagctcgagtggctctccctttaccctatccgttta | This study |
| ECA0785BamHI | gacaattggatccgacgcagtgaggcgctccccgctaccgcgttgatt | This study |
| ECA0785SacI | ttataaaagagctcagtgttttatttttatctgccagcgctgccccagc | This study |
| F-vasH-kompl | gacaattaagcttattccttatctgccgctggcgcatg | This study |
| R-vasH-kompl | ttataaaagagctccccataacgactgccaggcgggat | This study |
| Flp_pilin_compl_Fw | accaagcttaaccgcagttagtccgtc | This study |
| Flp_pilin_compl_Rv | aacgagctcgtactgatgcaccacagcaa | This study |
| **Sequencing primers for pMW119** | **Sequences** | **References** |
| pMW119(seq)_Fw | ctcactcattaggcacccca | Nykyri et al, 2012 |
| pMW119(seq)_Rv | gcctcttcgctattacgcca | Nykyri et al, 2012 |
| **Primers for qPCR** | **Sequences** | **References** |
| F_*proC* | aatgtcgcgggcaagct | Takle et al, 2007 |
| R_*proC* | tgaaaacgggcgatgttga | Takle et al, 2007 |
| F_hcp1 | tcagccatctggccagcg | Mattinen et al, 2007 |
| R_hcp1 | cttcagggttgtggtcgg | Mattinen et al, 2007 |
| F_hcp3 | cgaacgtgaccctgaaatg | Mattinen et al, 2007 |
| R_hcp3 | tggcgtcggtcagcacag | Mattinen et al, 2007 |
| Flp/FapF | gagcggtgtaacggcaatcgaata | This study |
| Flp/FapR | aaataccgtcgccgccgaaa | This study |
| F_ECA0789 | aggtagccagacaatcaacggtgt | This study |
| R_ECA0789 | tgcattaaccggctcgaccacata | This study |
| F_ECA0790 | aacaagccgattaacgatgtgcgg | This study |
| R_ECA0790 | ttgagctgtttcaaggattccggc | This study |
| F_ECA0792 | tcggatgactcggtcaacgacatt | This study |
| R_ECA0792 | taaaggtaatcggcgacagctcca | This study |
| F_ECA0029 | ttcccagttccacctgtcgattca | This study |
| R_ECA0029 | tcatggtattgaagcgggtagcca | This study |
| F_ECA1254 | agtggatgtccacgcgatttctca | This study |
| R_ECA1254 | caggccattgccaactggattcat | This study |
| F_ECA1264 | ttcggttgggtgagcgaagagaat | This study |
| R_ECA1264 | aactccagcttgaagtcgaccaga | This study |
| F_ECA1444 | gctcagccatttggcggtgataaa | This study |
| R_ECA1444 | agctgtgagaagccctgagcataa | This study |
| F_ECA2205 | aaaccgttgatgttgcctcatcgc | This study |
| R_ECA2205 | accagagcaatcgctgtcatcaga | This study |
| F_ECA2477 | ttatcgtgaatcctggcagccaca | This study |
| R_ECA2477 | aataacctcatcgccgcagacgaa | This study |
| F_ECA3258 | tgatcgatctgggtgcaggcaata | This study |
| R_ECA3258 | aacctgccaactgctctggactat | This study |
| F_ECA3929 | gcgcctgacgcattgatgaaagaa | This study |
| R_ECA3929 | taaggcaggattaacggacaggct | This study |
| F_ECA4044 | agcgcgcactatagtctggtgatt | This study |
| R_ECA4044 | ttgtcccgtgccgttaagatgagt | This study |
| F_ECA4116 | tattcaccgagctggttacgctgt | This study |
| R_ECA4116 | aggatctggctgttctgctgatgt | This study |
| F_ECA4464 | acctcgggatgtccggcattattt | This study |
| R_ECA4464 | acatacagcgcttgcttcatacgc | This study |

Amp^R^=ampicillin resistance, Cm^R^=chloramphenicol resistance
